# Supplementary material for: Estimation of Health Effects and Economic Losses from Ambient Air Pollution in Undeveloped Areas: Evidence from Guangxi, China
Source: Int J Environ Res Public Health. 2019 Jul 29;16(15):2707. doi: 10.3390/ijerph16152707 (PMC6695923; doi:10.3390/ijerph16152707)
Supplement: Supplementary file 1 [file ijerph-16-02707-s001.pdf]

Supplemental Materials

Article

Estimation of Health Effects and Economic Losses from Ambient Air Pollution in Undeveloped Areas: Evidence from Guangxi, China

Feng Han [1], Xingcheng Lu [2], Cuicui Xiao [3], Miao Chang [3,*] and Ke Huang [4]

[1] Key Laboratory of Beibu Gulf Environment Change and Resources Utilization of Ministry of Education, Nanning Normal University, Nanning 530001, China
[2] Division of Environment, Hong Kong University of Science & Technology, Clear Water Bay, Hong Kong, China
[3] School of Environmental, Tsinghua University, Beijing 100084, China
[4] Guangxi Environmental Protection Bureau, No.16 of Foziling Road, Nanning 530001, China
* Correspondence: changmiao@tsinghua.edu.cn; Tel.: +86-10-6278-0478; Fax: +86-10-62792747

Table S1. Concentration lower limit for ambient pollutants.

Pollutant	Concentration	Reference
SO_2	5 μg/m^3	WHO (2000)
NO_2	9.4 μg/m^3	WHO (2000)
PM_{10}	20 μg/m^3	WHO (2000)

Table S2. The Population of Municipal City of Guangxi Province (unit: 10,000 Person).

City	2010	2011	2012	2013	2014	2015	2016
NANNING	666.16	673.40	679.08	685.37	691.38	698.61	706.22
LIUZHOU	375.87	379.39	382.45	385.60	388.65	392.27	395.87
GUILIN	474.80	478.82	483.94	488.05	491.91	496.16	500.94
WUZHOU	288.22	290.85	292.94	295.44	297.55	299.94	301.84
BEIHAI	153.93	155.44	157.20	159.02	160.37	162.57	164.37
FANGCHENGGANG	86.69	87.84	88.69	89.90	90.80	91.84	92.90
QINZHOU	307.97	310.96	313.33	315.92	318.06	320.93	324.30
GUIGANG	411.88	415.67	418.68	422.05	425.56	429.37	433.20
YULIN	548.74	553.84	558.12	562.25	566.01	570.72	575.60
BAISE	346.68	349.46	351.81	354.52	356.88	359.67	362.02
HEZHOU	195.41	197.03	198.73	199.98	201.34	202.59	203.87
HECHI	336.93	339.34	341.55	343.19	345.14	347.68	349.90
LAIBING	209.97	211.82	213.51	214.90	216.37	218.20	220.05
CHONGZUO	199.43	201.14	201.97	202.81	203.98	205.45	206.92

Data Source: Statistical Bureau of Guangxi ZAR.

Table S3. CPI in China 2010–2015.

Year	2010	2011	2012	2013	2014	2015
Consumer Price Index (CPI)	3.33%	5.41%	2.64%	2.63%	2.00%	1.44%

Data Source: National Statistical Bureau of PRC.

Table S4. Hospital cost and impatient days of illness caused by ambient pollutants.

Cost of Medicine	Unit	2011	2012	2013	2014	2015	2016
Per capita outpatient expenses	RMB	131. 0	142.2	154.8	165.3	173.2	181
Per capita outpatient expenses	RMB	5,614. 0	6,013.6	6,527.0	6,835	7,154.6	7,544.2
Per capita impatient days	day	10.30	9.70	9.40	9.20	8.60	8.60

Data Source: China Health Statistics Yearbook.

Table S5. Socio-Economic Indicators of Municipal Cities in Guangxi in 2016.

CITY	Nanning	Liuzhou	Guilin	Wuzhou	Beihai	Fangchenggang	Qinzhou	Guigang	Yulin	Baise	Hezhou	Hechi	Laibing	Chongzuo
POLULATION (10,000 PERSON)	706.22	395.87	500.94	301.84	164.37	92.90	324.30	433.20	575.60	362.02	203.87	349.90	220.05	206.92
POPULATION DENSITY (10,000 PERSON/KM^2)	2.278	2.106	4.911	5.295	2.163	2.266	3.414	5.934	8.223	7.388	3.089	14.579	5.117	6.897
DEVELOPED AREA (KM^2)	310	188	102	57	76	41	95	73	70	49	66	24	43	30
URBAN FRACTION	57.77%	60.12%	44.57%	48.18%	53.64%	53.48%	35.51%	45.15%	44.77%	31.92%	40.76%	32.66%	38.56%	34.56%
NUMBER OF MOTOR VEHICLES	1042870	531578	487507	159161	207422	103128	718305	225610	400667	238999	132605	206898	136411	113578
COVERAGE OF GREEN AREA(HM^2)	41515.16	9181.22	4364.18	3279.12	3072.53	1382.07	12751.44	1682.8	2865	2171.58	1296.63	909.25	1503.54	1258
GROSS INDUSTRIAL PRODUCTION (100 MILLION RMB)	1063.14	1232.52	750.07	627.01	464.42	340.88	363.22	319.38	521.11	508.74	144.67	147.09	160.92	257.13
GDP (100 MILLION RMB)	3703.33	2476.94	2054.82	1175.65	1006.65	676.04	1102.05	958.76	1553.83	1114.31	518.19	657.18	589.11	766.20
INDUSTRY'S SHARE OF GDP	28.71%	49.76%	36.50%	53.33%	46.14%	50.42%	32.96%	33.31%	33.54%	45.66%	27.92%	22.38%	27.32%	33.56%
EXPENDITURE FOR ENERGY CONSERVATION & ENVIRONMENT PROTECTION (10,000 RMB YUAN)	140880	38595	21050	5297	12987	10995	11417	8237	33984	28129	14801	59474	9620	9056
INDUSTRIAL ENERGY CONSUMPTION (TONS OF STANDARD COAL.)	2966.24	6515.66	2066.61	925.93	1415.92	2711.92	2521.33	3003.76	1476.76	4477.63	1118.36	897.34	2536.05	1804.55
VEHICLES OWNED PERCAPITA	0.148	0.134	0.097	0.053	0.126	0.111	0.221	0.052	0.070	0.066	0.065	0.059	0.062	0.055
RATE OF ENVIROMENT COST IN GDP	0.38%	0.16%	0.10%	0.05%	0.13%	0.16%	0.10%	0.09%	0.22%	0.25%	0.29%	0.90%	0.16%	0.12%
RESIDENTIAL INCOME (RMB YUAN/YEAR)	52723	62855	41216	39072	61580	73188	34160	22230	27110	30881	25498	18842	26885	37161

Data Source: Statistical Bureau of Guangxi ZAR.

Table S6. Monthly average concentration of SO_2 in 14 cities over Guangxi ZAR.

Month	BAISE	BEIHAI	CHONGZUO	FANGCHENGGANG	GUIGANG	GUILIN	HECHI	HEZHOU	LAIBING	LIUZHOU	NANNING	QINZHOU	WUZHOU	YULIN
Jan-11	34.9	28.4	26.7	25.6	18.3	42.5	30.3	10.1	38.5	80.0	38.6	32.7	23.4	33.7
Feb-11	29.8	20.2	27.0	14.1	23.3	33.0	22.3	11.4	37.7	45.3	22.3	19.6	20.5	36.5
Mar-11	49.0	22.2	27.3	14.4	11.9	34.5	59.3	14.8	28.0	93.5	21.1	21.5	23.6	32.2
Apr-11	51.6	19.0	18.0	13.2	14.6	36.1	48.2	19.5	38.6	80.7	19.3	17.3	36.0	56.2
May-11	69.9	20.7	14.1	15.5	28.8	23.9	45.8	18.9	32.4	60.4	18.5	19.3	26.1	43.1
Jun-11	33.9	17.5	17.6	11.7	20.4	25.3	35.6	20.0	21.3	46.5	13.7	14.5	24.2	51.8
Jul-11	38.7	19.7	25.5	8.8	15.2	28.7	34.6	16.4	21.2	48.3	16.2	11.5	24.6	45.4

Month	BAISE	BEIHAI	CHONGZUO	FANGCHENGGANG	GUIGANG	GUILIN	HECHI	HEZHOU	LAIBING	LIUZHOU	NANNING	QINZHOU	WUZHOU	YULIN
Aug-11	61.4	19.9	25.4	11.0	10.4	27.3	43.4	14.3	31.6	36.0	20.5	9.5	27.0	40.3
Sep-11	60.1	23.5	33.9	10.1	15.2	30.1	39.5	17.6	36.4	46.1	21.9	11.5	22.7	37.2
Oct-11	58.4	24.5	21.5	10.4	18.1	28.7	53.0	16.2	25.5	70.7	22.7	18.4	20.4	29.6
Nov-11	65.2	25.7	22.8	15.3	22.7	53.4	72.1	16.9	29.3	77.3	37.8	22.7	25.5	48.4
Dec-11	58.8	35.4	23.8	30.0	25.1	59.3	55.3	19.1	25.7	81.3	55.9	30.8	34.4	31.5
Jan-12	16.2	19.5	29.2	10.3	16.3	21.0	15.1	16.2	42.4	45.6	18.9	21.2	16.2	13.3
Feb-12	13.8	21.6	21.3	11.7	13.1	25.0	18.9	16.2	58.2	56.7	19.6	21.6	15.7	24.1
Mar-12	15.5	21.2	18.8	6.0	19.7	35.4	17.8	16.9	44.7	59.1	21.4	20.3	13.6	46.5
Apr-12	15.9	18.5	19.4	7.1	18.1	26.6	15.9	14.9	48.5	45.6	18.3	20.7	10.3	48.7
May-12	12.5	13.5	19.2	8.2	14.8	18.3	22.9	12.3	55.8	41.9	11.1	13.5	10.8	49.0
Jun-12	10.6	15.6	20.5	7.7	13.8	19.3	22.5	13.5	47.7	40.8	13.8	10.9	10.2	49.0
Jul-12	11.2	16.2	11.9	9.5	14.5	12.9	20.6	12.7	41.2	23.7	11.0	13.6	9.5	44.9
Aug-12	11.6	16.4	24.3	6.1	13.6	12.7	24.7	14.6	42.3	42.9	17.5	17.5	17.1	53.7
Sep-12	13.0	20.0	25.0	7.5	12.4	16.1	19.2	13.7	56.5	45.8	23.3	20.1	13.8	47.3
Oct-12	12.2	18.1	26.5	8.7	20.2	28.9	23.2	17.3	44.4	51.6	24.6	17.5	18.9	50.3
Nov-12	14.6	18.8	32.8	9.2	22.1	26.2	22.8	17.1	47.2	61.2	23.9	18.3	17.4	48.8
Dec-12	17.8	19.2	22.4	13.7	26.1	34.8	21.5	15.6	47.1	70.9	27.3	19.5	16.2	47.7
Jan-13	67.2	16.7	19.6	11.9	25.1	46.5	33.5	16.8	17.7	52.7	26.3	24.3	16.8	50.3
Feb-13	39.5	15.1	13.8	7.9	17.8	12.0	17.0	15.4	15.6	30.5	12.4	17.6	10.5	33.8
Mar-13	39.7	18.9	17.5	9.8	19.6	30.2	14.2	18.4	16.5	45.2	18.4	18.6	12.1	57.0
Apr-13	48.1	20.8	17.9	8.8	11.2	25.9	17.5	18.4	15.0	31.6	14.3	18.3	10.9	44.4
May-13	42.5	18.2	8.5	8.0	12.0	18.0	18.3	19.7	14.1	25.6	11.5	17.4	14.8	54.4
Jun-13	57.8	13.9	8.4	9.5	9.6	21.5	20.7	22.8	17.7	20.8	12.5	12.4	18.8	46.8
Jul-13	46.4	10.6	10.3	6.5	10.6	20.4	14.3	29.2	50.4	15.2	10.5	12.6	15.2	50.4
Aug-13	44.9	14.5	10.4	5.9	9.0	19.8	18.1	26.2	31.1	23.4	11.5	13.4	15.0	54.0
Sep-13	29.5	17.2	15.5	8.6	14.3	21.3	34.2	20.7	24.5	27.1	16.0	16.3	15.9	52.3
Oct-13	35.2	20.5	18.9	10.8	20.7	35.5	52.4	27.4	28.4	40.9	30.1	19.2	22.6	50.2
Nov-13	55.2	-	15.1	9.7	16.1	32.5	36.7	14.7	29.4	39.5	25.9	15.3	22.0	39.9
Dec-13	60.5	28.4	21.7	18.9	16.4	43.7	36.8	18.4	38.6	59.6	39.8	21.0	19.8	48.9
Jan-14	76.6	20.2	16.6	-	-	44.8	33.7	42.2	40.1	46.7	31.1	-	22.0	-
Feb-14	61.2	10.1	5.8	-	-	15.0	10.3	16.1	18.8	22.3	13.7	-	29.1	-
Mar-14	85.9	15.3	6.7	-	-	17.6	14.3	21.7	19.2	30.7	12.9	-	20.3	-
Apr-14	58.0	15.0	10.1	-	-	18.2	8.4	18.6	19.5	24.8	11.3	-	30.3	-
May-14	45.3	16.0	11.5	-	-	15.7	11.5	65.6	13.0	26.4	10.8	-	44.0	-
Jun-14	23.6	11.9	9.5	-	-	10.9	7.9	95.9	17.2	23.1	11.1	-	17.0	-
Jul-14	35.5	12.4	16.3	-	-	15.3	8.1	11.9	26.4	22.0	10.6	-	21.1	-
Aug-14	29.9	11.7	13.6	-	-	11.8	6.1	11.2	28.2	21.8	10.1	-	21.6	-
Sep-14	36.5	7.3	21.0	-	-	19.3	6.9	32.4	26.5	28.2	11.2	-	27.0	-
Oct-14	55.3	12.1	30.4	-	-	29.8	7.9	21.4	31.7	40.0	16.3	-	37.8	-
Nov-14	27.4	11.3	21.6	-	-	26.8	11.3	26.1	24.6	41.7	14.7	-	39.8	-
Dec-14	28.3	17.6	19.7	16.8	17.2	40.0	31.9	17.1	25.2	49.8	24.6	31.9	30.7	43.1
Jan-15	23.3	12.1	17.6	10.4	16.1	29.1	25.6	18.1	32.4	37.1	18.9	22.2	28.7	70.5
Feb-15	25.6	7.4	7.3	7.6	17.7	18.3	15.0	10.4	22.4	24.5	14.0	18.6	23.0	27.6
Mar-15	18.9	6.5	7.2	6.1	19.2	16.3	19.6	11.5	13.4	17.5	10.1	15.6	21.7	36.8
Apr-15	26.3	6.4	9.0	4.8	21.2	20.0	16.0	18.4	28.2	22.0	13.3	17.1	21.7	52.6

Month	BAISE	BEIHAI	CHONGZUO	FANGCHENGGANG	GUIGANG	GUILIN	HECHI	HEZHOU	LAIBING	LIUZHOU	NANNING	QINZHOU	WUZHOU	YULIN
May-15	14.6	5.8	6.3	4.2	22.8	16.5	17.0	14.9	17.1	18.9	9.8	10.3	11.1	43.3
Jun-15	15.0	7.5	8.0	6.7	25.3	14.9	7.0	13.7	22.5	15.7	8.6	10.8	13.6	23.7
Jul-15	11.5	8.5	9.2	5.9	19.5	17.6	23.7	16.7	17.0	19.1	10.9	14.1	17.7	15.3
Aug-15	9.6	9.0	11.3	4.2	21.1	20.7	42.6	17.6	23.0	23.8	12.8	14.6	17.3	17.4
Sep-15	11.9	8.4	10.7	5.0	17.9	23.0	28.7	17.6	20.3	24.3	11.3	14.8	13.3	25.9
Oct-15	14.9	12.0	18.4	4.7	24.1	28.9	44.7	24.0	21.2	33.7	17.6	24.0	16.2	28.3
Nov-15	14.7	10.3	12.3	5.3	31.6	21.1	30.0	19.3	20.7	26.6	12.8	20.8	12.8	34.7
Dec-15	14.8	11.1	10.4	4.3	27.7	20.8	24.5	16.4	17.9	31.0	14.8	25.3	13.2	16.2
Jan-16	13.4	9.2	9.3	4.3	26.5	17.5	23.9	16.0	19.2	28.3	13.1	23.9	11.1	19.7
Feb-16	10.6	8.6	9.7	4.6	27.9	16.3	25.1	17.9	30.0	28.9	16.0	20.2	17.1	18.2
Mar-16	12.8	8.6	10.3	5.7	22.6	21.5	10.2	20.7	31.9	24.0	12.5	17.9	14.5	34.0
Apr-16	12.9	7.6	9.2	8.0	18.5	19.1	7.7	19.5	18.2	19.5	9.5	17.8	8.8	41.5
May-16	10.3	7.4	9.5	12.7	26.6	19.1	5.7	21.2	20.1	18.0	10.0	16.7	7.2	25.0
Jun-16	7.3	6.2	10.0	12.5	38.2	12.1	6.5	22.0	30.7	12.6	8.3	11.8	9.5	21.8
Jul-16	10.9	7.9	10.6	8.5	16.0	14.1	6.5	22.5	33.6	11.8	8.8	11.4	9.9	22.5
Aug-16	6.5	8.1	8.3	6.7	11.3	18.4	9.3	19.2	20.4	18.9	10.4	13.6	8.8	28.9
Sep-16	11.4	10.7	13.6	8.7	14.2	20.8	14.0	14.7	17.7	26.5	13.9	18.7	11.8	23.0
Oct-16	15.0	12.5	16.6	10.9	14.5	14.8	12.3	13.6	17.5	21.3	14.9	18.8	11.2	17.6

Data Source: Guangxi Environmental Monitoring Station.

Table S7. Adverse Health Effect by PM_{10}.

		All-Cause Mortality	Cardiovascular Mortality	Respiratory Mortality	All-Caused Hospital Admission	Cardiovascular Impatient	Respiratory Impatient	ALL CAUSED Outpatient Visits
Total		2,8396(1,4664–4,2014)	1,5500(8,144–2,2437)	5,558(3,096–7,991)	4,18159(2,75591–5,59898)	1,5722(−2,063–3,2617)	4,7083(3,3197–6,6748)	1,6397,047(0–3,2595,751)
Nanning		5,047(2,608–7,462)	2,752(1,447–3,980)	987(550–1,417)	7,4068(4,8840–9,9125)	2,751(−361–5,702)	8,279(5,842–1,1722)	2,912747(0–5,784235)
	2011	778(402–1,151)	403(212–583)	148(82–212)	8,678(5,721–1,1616)	345(−45–716)	1,104(779–1,563)	4,12369(0–8,19313)
	2012	793(410–1,172)	397(209–574)	177(98–254)	1,0335(6,813–1,3834)	304(−40–631)	933(658–1,322)	4,54511(0–9,03042)
	2013	1,058(547–1,562)	583(307–842)	201(112–288)	1,5432(1,0184–2,0637)	428(−56–885)	1,281(905–1,809)	6,09658(0–1,208728)
	2014	977(505–1,443)	547(288–790)	186(104–268)	1,5368(1,0138–2,0558)	519(−68–1,074)	1,529(1,080–2,162)	5,75323(0–1,141532)
	2015	806(416–1,192)	452(238–655)	152(85–219)	1,3018(8,581–1,7428)	581(−76–1,205)	1,713(1,209–2,427)	4,71957(0–9,37843)
	2016	636(328–941)	370(194–536)	123(68–177)	1,1237(7,403–1,5052)	574(−75–1,191)	1,720(1,212–2,440)	3,88929(0–7,73778)
Guilin		3,569(1,844–5,276)	1,943(1,022–2,811)	699(390–1,004)	5,2220(3,4432–6,9889)	1,943(−255–4,028)	5,845(4,124–8,276)	2,058441(0–4,088049)
	2011	583(301–862)	302(159–437)	110(62–159)	6,499(4,285–8,698)	259(−34–536)	826(583–1,169)	3,08875(0–6,13470)
	2012	595(307–880)	298(157–431)	133(74–190)	7,757(5,115–1,0382)	228(−30–474)	691(487–979)	3,41203(0–6,77677)
	2013	685(354–1,012)	378(199–546)	130(73–186)	9,992(6,591–1,3367)	277(−36–573)	830(586–1,174)	3,94604(0–7,82979)
	2014	718(371–1,060)	402(212–581)	137(76–197)	1,1293(7,450–1,5105)	381(−50–789)	1,123(794–1,587)	4,22814(0–8,38708)
	2015	517(267–765)	290(152–420)	98(54–140)	8,345(5,499–1,1175)	372(−49–772)	1,099(775–1,559)	3,02468(0–6,01425)
	2016	471(243–698)	274(144–397)	91(51–131)	8,334(5,491–1,1162)	425(−56–883)	1,275(899–1,808)	2,88477(0–5,73790)
Liuzhou		3,086(1,595–4,562)	1,686(887–2,438)	603(336–865)	4,5545(3,0037–6,0943)	1,688(−222–3,496)	4,841(3,417–6,851)	1,784521(0–3,542637)
	2011	450(233–666)	233(123–337)	85(48–123)	5,019(3,309–6,718)	200(−26–414)	638(450–904)	2,38532(0–4,73842)
	2012	458(237–678)	229(121–332)	102(57–147)	5,975(3,939–7,998)	176(−23–365)	556(392–788)	2,62813(0–5,22075)
	2013	578(299–854)	318(168–460)	110(61–157)	8,430(5,563–1,1275)	234(−31–483)	700(495–989)	3,33006(0–6,60396)
	2014	774(400–1,143)	433(228–625)	148(82–212)	1,2175(8,035–1,6280)	411(−54–851)	963(681–1,360)	4,55979(0–9,03847)

		All-Cause Mortality	Cardiovascular Mortality	Respiratory Mortality	All-Caused Hospital Admission	Cardiovascular Impatient	Respiratory Impatient	ALL CAUSED Outpatient Visits
	2015	434(224–642)	244(128–353)	82(46–118)	7,011(4,621–9,387)	313(−41–649)	923(651–1,308)	2,54150(0–5,05165)
	2016	392(203–580)	228(120–330)	76(42–109)	6,934(4,569–9,285)	354(−46–735)	1,060(747–1,503)	2,40042(0–4,77311)
Guigang		2,694(1,391–3,986)	1,467(771–2,124)	528(294–759)	3,9371(2,5948–5,2714)	1,459(−192–3,028)	4,478(3,158–6,348)	1,552603(0–3,086208)
	2011	438(226–648)	227(119–329)	83(46–119)	4,884(3,219–6,540)	194(−26–403)	622(438–881)	2,32037(0–4,61292)
	2012	446(230–659)	223(117–323)	99(55–143)	5,810(3,829–7,779)	171(−22–355)	599(422–848)	2,55447(0–5,07831)
	2013	549(284–811)	302(159–437)	104(58–150)	8,003(5,278–1,0709)	222(−29–459)	666(470–942)	3,15974(0–6,27329)
	2014	509(263–753)	285(150–413)	97(54–140)	8,010(5,281–1,0722)	270(−36–560)	799(564–1,131)	2,99725(0–5,95432)
	2015	425(219–629)	239(125–346)	80(45–116)	6,863(4,522–9,192)	306(−40–635)	905(638–1,283)	2,48726(0–4,94708)
	2016	328(169–486)	191(100–277)	64(35–91)	5,800(3,820–7,773)	296(−39–615)	889(626–1,263)	2,00693(0–3,99616)
Yulin		2,419(1,247–3,584)	1,323(694–1,919)	475(264–684)	3,5904(2,3641–4,8117)	1,397(−183–2,903)	4,112(2,895–5,843)	1,398455(0–2,785349)
	2011	384(198–570)	199(105–289)	73(41–105)	4,287(2,822–5,746)	170(−22–354)	548(385–778)	2,03495(0–4,05384)
	2012	391(202–580)	196(103–284)	87(49–126)	5,102(3,359–6,838)	150(−20–312)	351(247–500)	2,24134(0–4,46499)
	2013	288(148–427)	159(83–231)	55(30–79)	4,200(2,764–5,632)	116(−15–241)	352(247–501)	1,65531(0–3,30110)
	2014	474(245–702)	266(140–385)	91(50–131)	7,462(4,915–9,998)	252(−33–522)	747(526–1,060)	2,78985(0–5,55353)
	2015	473(244–700)	266(139–385)	89(50–129)	7,637(5,030–1,0232)	340(−45–707)	1,008(710–1,431)	2,76652(0–5,50750)
	2016	408(211–605)	238(125–344)	79(44–114)	7,216(4,752–9,671)	368(−48–765)	1,106(779–1,572)	2,49658(0–4,97253)
Baise		2,181(1,126–3,227)	1,196(628–1,731)	426(237–612)	3,2557(2,1459–4,3589)	1,241(−163–2,573)	3,690(2,603–5,230)	1,263889(0–2,512170)
	2011	300(155–444)	155(82–225)	57(32–82)	3,344(2,202–4,480)	133(−17–276)	426(300–605)	1,58779(0–3,16007)
	2012	305(157–452)	153(80–221)	68(38–98)	3,975(2,618–5,326)	117(−15–243)	321(226–456)	1,74708(0–3,47711)
	2013	304(157–450)	168(88–243)	58(32–83)	4,433(2,920–5,939)	123(−16–255)	370(261–526)	1,74862(0–3,48040)
	2014	568(294–839)	318(167–459)	108(61–155)	8,937(5,898–1,1950)	302(−40–624)	888(628–1,253)	3,34717(0–6,63456)
	2015	381(197–564)	214(112–310)	72(40–104)	6,155(4,057–8,242)	275(−36–570)	811(572–1,149)	2,23110(0–4,43585)
	2016	323(167–478)	188(99–272)	63(35–90)	5,713(3,763–7,652)	292(−38–605)	874(616–1,240)	1,97714(0–3,93371)
Hechi		1,971(1,017–2,917)	1,074(564–1,556)	386(215–556)	2,8911(1,9049–3,8723)	1,095(−144–2,273)	3,343(2,356–4,742)	1,136162(0–2,259976)
	2011	324(167–479)	168(88–243)	61(34–88)	3,612(2,380–4,838)	144(−19–298)	460(324–653)	1,71557(0–3,41251)
	2012	329(170–487)	165(87–239)	73(41–106)	4,293(2,829–5,750)	126(−17–262)	424(299–601)	1,88729(0–3,75408)
	2013	344(178–510)	190(100–275)	65(36–94)	5,024(3,311–6,729)	139(−18–288)	419(295–594)	1,98246(0–3,94255)
	2014	332(171–491)	186(98–269)	63(35–91)	5,218(3,438–6,989)	176(−23–365)	521(367–740)	1,95141(0–3,88182)
	2015	378(195–559)	212(112–307)	71(40–103)	6,107(4,025–8,177)	272(−36–565)	804(567–1,140)	2,21369(0–4,40054)
	2016	263(136–390)	153(80–222)	51(28–73)	4,657(3,067–6,240)	238(−31–494)	714(503–1,014)	1,61119(0–3,20826)
Qinzhou		1,656(855–2,452)	901(473–1,305)	326(181–469)	2,4125(1,5891–3,2320)	911(−119–1,893)	2,744(1,933–3,895)	9,53269(0–1,897098)
	2011	296(153–439)	154(81–222)	56(31–81)	3,304(2,177–4,426)	131(−17–273)	421(297–597)	1,56951(0–3,12200)
	2012	302(156–446)	151(79–219)	67(37–97)	3,932(2,591–5,266)	116(−15–240)	339(239–481)	1,72851(0–3,43827)
	2013	298(154–441)	164(86–238)	57(32–81)	4,345(2,863–5,820)	120(−16–250)	363(256–514)	1,71433(0–3,41050)
	2014	248(128–367)	139(73–201)	47(26–68)	3,901(2,569–5,228)	131(−17–273)	391(275–555)	1,45844(0–2,90416)
	2015	273(141–405)	154(81–223)	52(29–74)	4,416(2,908–5,916)	197(−26–409)	583(410–827)	1,59971(0–3,18424)
	2016	239(123–354)	139(73–202)	46(26–67)	4,226(2,783–5,663)	216(−28–448)	648(456–920)	1,46219(0–2,91181)
Wuzhou		1,311(676–1,942)	724(380–1,049)	255(142–367)	1,9895(1,3103–2,6658)	770(−101–1,599)	2,378(1,675–3,376)	7,62523(0–1,518183)
	2011	144(74–213)	75(39–108)	27(15–39)	1,604(1,055–2,151)	64(−8–133)	205(144–292)	7,6087(0–1,51747)
	2012	146(75–217)	73(38–106)	33(18–47)	1,907(1,255–2,558)	56(−7–117)	234(165–333)	8,3759(0–1,67047)
	2013	214(110–317)	118(62–171)	41(23–59)	3,119(2,054–4,180)	86(−11–179)	261(184–371)	1,22991(0–2,44981)
	2014	323(167–477)	181(95–262)	62(34–89)	5,074(3,344–6,794)	171(−22–355)	506(357–718)	1,89816(0–3,77326)
	2015	245(127–363)	138(72–200)	46(26–67)	3,964(2,611–5,312)	177(−23–367)	523(369–743)	1,43608(0–2,85907)
	2016	239(123–354)	139(73–202)	46(26–67)	4,227(2,784–5,664)	216(−28–448)	648(456–919)	1,46262(0–2,91174)

	All-Cause Mortality	Cardiovascular Mortality	Respiratory Mortality	All-Caused Hospital Admission	Cardiovascular Impatient	Respiratory Impatient	ALL CAUSED Outpatient Visits
Laibing	1,312(677–1,942)	712(374–1,031)	258(144–372)	1,9047(1,2552–2,5506)	720(−94–1,494)	2,093(1,476–2,969)	7,55003(0–1,501123)
2011	250(129–370)	130(68–187)	47(26–68)	2,790(1,839–3,734)	111(−15–230)	355(250–502)	1,32564(0–2,63345)
2012	255(132–377)	127(67–184)	57(32–82)	3,321(2,189–4,445)	98(−13–203)	216(152–307)	1,46045(0–2,90126)
2013	170(88–252)	94(49–136)	32(18–47)	2,486(1,637–3,331)	69(−9–143)	208(146–295)	9,8035(0–1,95197)
2014	259(134–383)	145(76–210)	49(28–71)	4,071(2,684–5,449)	137(−18–285)	406(286–575)	1,52325(0–3,02608)
2015	199(103–295)	112(59–162)	38(21–54)	3,214(2,117–4,306)	143(−19–298)	424(299–602)	1,16466(0–2,31749)
2016	179(92–265)	104(55–151)	35(19–50)	3,166(2,085–4,242)	162(−21–336)	485(342–689)	1,09569(0–2,18098)
Chongzuo	1,011(522–1,498)	551(289–799)	198(110–286)	1,4827(9,765–1,9865)	556(−73–1,155)	1,685(1,187–2,392)	5,82996(0–1,160486)
2011	165(85–245)	86(45–124)	31(17–45)	1,846(1,216–2,473)	73(−10–153)	236(166–334)	8,7643(0–1,74466)
2012	168(87–249)	84(44–122)	37(21–54)	2,189(1,442–2,933)	64(−8–134)	200(141–285)	9,6187(0–1,91474)
2013	182(94–269)	100(53–145)	35(19–50)	2,652(1,747–3,552)	73(−10–152)	221(156–314)	1,04598(0–2,08142)
2014	195(101–289)	109(57–159)	37(21–54)	3,073(2,024–4,115)	104(−14–215)	307(216–436)	1,14908(0–2,28583)
2015	165(85–244)	93(49–134)	31(17–45)	2,662(1,753–3,567)	119(−16–247)	352(248–499)	9,6434(0–1,92006)
2016	136(70–202)	79(42–115)	26(15–38)	2,406(1,584–3,225)	123(−16–255)	369(260–525)	8,3226(0–1,65815)
Hezhou	992(512–1,468)	549(288–794)	192(107–276)	1,5162(9,992–2,0302)	579(−76–1,201)	1,770(1,248–2,509)	5,78233(0–1,149684)
2011	89(46–132)	46(24–67)	17(9–24)	995(654–1,334)	40(−5–82)	127(90–181)	4,7186(0–9,4130)
2012	91(47–135)	46(24–66)	20(11–29)	1,185(779–1,589)	35(−5–72)	144(101–205)	5,2018(0–1,03769)
2013	138(71–205)	76(40–111)	26(15–38)	2015(1,327–2,701)	56(−7–116)	169(119–240)	7,9458(0–1,58300)
2014	339(175–500)	190(100–274)	65(36–93)	5,333(3,521–7,129)	180(−24–372)	529(374–747)	1,99774(0–3,95774)
2015	184(95–272)	103(54–149)	35(19–50)	2,964(1,953–3,971)	132(−17–274)	391(276–555)	1,07406(0–2,13727)
2016	151(78–224)	88(46–127)	29(16–42)	2,670(1,758–3,579)	136(−18–283)	409(288–581)	9,2391(0–1,83984)
Beihai	718(370–1,064)	389(204–564)	142(79–204)	1,0370(6,829–1,3897)	385(−50–800)	1,151(810–1,635)	4,12345(0–8,21106)
2011	137(71–202)	71(37–103)	26(14–37)	1,525(1,004–2,043)	61(−8–126)	194(137–276)	7,2406(0–1,44087)
2012	140(72–207)	70(37–101)	31(17–45)	1,821(1,200–2,439)	54(−7–111)	147(103–209)	8,0034(0–1,59267)
2013	117(60–174)	65(34–94)	22(12–32)	1,711(1,126–2,293)	47(−6–98)	143(101–203)	6,7456(0–1,34353)
2014	136(70–201)	76(40–110)	26(14–37)	2,134(1,406–2,859)	72(−9–149)	213(150–303)	7,9786(0–1,58816)
2015	103(53–152)	58(30–84)	19(11–28)	1,661(1,093–2,227)	74(−10–154)	220(155–312)	6,0144(0–1,19865)
2016	86(44–127)	50(26–73)	17(9–24)	1,519(999–2,036)	77(−10–161)	233(164–332)	5,2519(0–1,04718)
Fangchenggang	429(221–635)	232(122–336)	85(47–122)	6,156(4,054–8,248)	228(−30–473)	675(475–959)	2,45859(0–4,89447)
2011	85(44–126)	44(23–64)	16(9–23)	951(627–1,274)	38(−5–79)	121(85–172)	4,5192(0–8,9885)
2012	87(45–129)	44(23–63)	19(11–28)	1,134(747–1,519)	33(−4–69)	86(60–122)	4,9872(0–9,9193)
2013	76(39–113)	42(22–61)	15(8–21)	1,112(733–1,490)	31(−4–64)	93(65–132)	4,3873(0–8,7329)
2014	69(35–102)	39(20–56)	13(7–19)	1,082(712–1,449)	36(−5–76)	108(76–154)	4,0428(0–8,0514)
2015	61(32–91)	35(18–50)	12(6–17)	992(653–1,330)	44(−6–92)	131(92–186)	3,5919(0–7,1570)
2016	50(26–74)	29(15–42)	10(5–14)	884(582–1,185)	45(−6–94)	136(96–193)	3,0574(0–6,0956)

Table S8. Adverse Health Effect caused by SO.

		**All-Cause Mortality**	**Cardiovascular Mortality**	**Respiratory Mortality**	**All-Caused Hospital Admission**	**Cardiovascular Impatient**	**Respiratory Impatient**	**ALL CAUSED Outpatient Visits**
Total		2,4618(1,5480–3,3371)	1,1749(6,682–1,6772)	5,004(3,140–6,886)	5,48136(4,14829–6,75991)	1,7091(5,022–2,9292)	4,6483(2,8332–6,4380)	1,6518,440(7,92306–3,2018,916)
Yulin		4,197(2,641–5,684)	2,026(1,154–2,889)	839(527–1,153)	9,5587(7,2411–1,17772)	3,027(891–5,181)	4,059(2,469–5,634)	2,829677(1,35991–5,474286)
	2011	914(575–1,237)	417(238–594)	180(113–247)	1,5910(1,2062–1,9589)	534(157–913)	541(329–750)	5,69113(2,7390–1,099439)
	2012	260(163–354)	115(65–165)	61(38–84)	5,328(4,025–6,584)	131(39–226)	347(211–482)	1,75172(8,366–3,40997)
	2013	1,154(728–1,561)	561(320–798)	227(143–311)	2,6247(1,9921–3,2279)	614(181–1,047)	347(211–483)	7,81594(3,7708–1,506237)
	2014	601(378–815)	297(169–424)	120(75–165)	1,4816(1,1211–1,8274)	420(123–720)	737(449–1,022)	4,15799(1,9933–8,06390)
	2015	745(469–1,010)	369(210–526)	146(92–201)	1,8832(1,4260–2,3212)	707(208–1,211)	995(606–1,380)	5,12817(2,4621–9,93056)
	2016	522(328–708)	268(152–382)	105(66–145)	1,4454(1,0933–1,7835)	620(182–1,064)	1,092(664–1,516)	3,75182(1,7972–7,28167)
Liuzhou		3,136(1,975–4,245)	1,489(849–2,122)	634(399–870)	6,8102(5,1610–8,3878)	2,140(630–3,658)	4,779(2,917–6,610)	2,089767(1,00552–4,038115)
	2011	1,033(653–1,394)	471(270–668)	202(128–276)	1,7901(1,3616–2,1969)	605(179–1,028)	630(384–872)	6,44412(3,1233–1,236261)
	2012	420(264–570)	186(105–265)	98(61–134)	8,580(6,493–1,0582)	212(62–364)	549(335–760)	2,83106(1,3576–5,48900)
	2013	536(337–726)	261(148–372)	106(67–146)	1,2225(9,260–1,5065)	285(84–488)	691(422–954)	3,62648(1,7422–7,01834)
	2014	486(306–658)	240(136–342)	97(61–133)	1,1960(9,055–1,4743)	340(100–581)	951(582–1,312)	3,36015(1,6128–6,50886)
	2015	364(229–494)	180(102–258)	72(45–99)	9,218(6,972–1,1375)	346(101–593)	911(556–1,262)	2,50474(1,1997–4,86189)
	2016	297(186–402)	152(86–217)	60(37–82)	8,218(6,213–1,0145)	352(103–605)	1,047(638–1,450)	2,13112(1,0198–4,14044)
Guilin		2,760(1,735–3,743)	1,314(747–1,877)	564(354–776)	6,1324(4,6391–7,5657)	1,909(561–3,274)	5,770(3,521–7,984)	1,851547(8,8732–3,592049)
	2011	675(425–914)	308(176–440)	133(84–183)	1,1771(8,917–1,4502)	395(116–675)	816(498–1,128)	4,20432(2,0203–8,13468)
	2012	486(305–659)	214(122–306)	113(71–155)	9,919(7,504–1,2236)	245(72–421)	682(416–944)	3,27092(1,5675–6,34567)
	2013	518(326–703)	252(143–360)	103(64–141)	1,1839(8,958–1,4603)	275(81–472)	820(501–1,133)	3,50513(1,6804–6,79764)
	2014	398(250–540)	197(112–281)	79(50–109)	9,822(7,426–1,2123)	278(82–477)	1,109(678–1,532)	2,75238(1,3174–5,34644)
	2015	401(252–544)	198(113–284)	79(49–109)	1,0149(7,674–1,2528)	380(112–652)	1,085(662–1,503)	2,75590(1,3190–5,35347)
	2016	282(177–383)	145(82–207)	57(36–79)	7,825(5,912–9,665)	335(98–576)	1,259(767–1,744)	2,02681(9,687–3,94259)
Nanning		2,693(1,691–3,654)	1,268(720–1,813)	560(351–772)	5,8673(4,4354–7,2437)	1,770(519–3,039)	8,174(4,988–1,1308)	1,801222(8,6193–3,499543)
	2011	654(411–887)	299(170–427)	129(81–178)	1,1431(8,647–1,4102)	382(112–656)	1,090(665–1,508)	4,07243(1,9514–7,90173)
	2012	742(466–1,006)	327(186–467)	172(108–237)	1,5142(1,1459–1,8675)	375(110–643)	921(561–1,275)	4,99601(2,3956–9,68693)
	2013	462(290–627)	225(128–321)	92(57–126)	1,0571(7,989–1,3053)	245(72–421)	1,264(773–1,745)	3,12254(1,4933–6,07056)
	2014	327(205–444)	161(92–231)	65(41–90)	8,067(6,093–9,967)	228(67–392)	1,510(923–2,086)	2,25630(1,0776–4,39212)
	2015	266(167–362)	132(75–189)	53(33–73)	6,752(5,098–8,344)	252(74–434)	1,691(1,032–2,341)	1,82880(8,729–3,56205)
	2016	242(152–328)	124(70–178)	49(31–67)	6,711(5,067–8,295)	287(84–494)	1,698(1,034–2,353)	1,73614(8,286–3,38206)
Baise		2,691(1,695–3,641)	1,287(733–1,832)	539(339–739)	5,9132(4,4831–7,2801)	1,752(516–2,994)	3,643(2,221–5,045)	1,798808(8,6599–3,474006)
	2011	747(471–1,010)	341(195–485)	147(93–201)	1,2988(9,861–1,5967)	437(129–746)	421(256–584)	4,65921(2,2495–8,97241)
	2012	213(134–289)	94(53–134)	50(31–68)	4,351(3,288–5,374)	107(31–185)	317(193–439)	1,43144(6,842–2,78407)
	2013	705(444–953)	342(195–487)	139(87–190)	1,6033(1,2167–1,9722)	375(111–640)	366(223–507)	4,77269(2,3017–9,20142)
	2014	694(437–938)	342(195–487)	137(87–188)	1,7025(1,2917–2,0945)	485(143–828)	877(536–1,210)	4,80265(2,3154–9,26216)
	2015	197(124–268)	98(55–140)	39(24–54)	4,996(3,775–6,172)	187(55–321)	800(488–1,109)	1,35466(6,473–2,63578)
	2016	135(84–183)	69(39–99)	27(17–38)	3,739(2,823–4,621)	160(47–275)	863(526–1,196)	9,6743(4,618–1,88421)
Hechi		1,820(1,145–2,467)	860(489–1,227)	370(232–509)	3,9014(2,9525–4,8114)	1,223(359–2,096)	3,300(2010–4,573)	1,206714(5,7888–2,338728)
	2011	632(398–855)	289(165–411)	124(78–170)	1,0997(8,343–1,3532)	370(109–631)	454(277–630)	3,93873(1,8982–7,59855)
	2012	293(184–398)	130(74–185)	68(43–94)	5,995(4,533–7,399)	148(44–254)	419(255–580)	1,97526(9,457–3,83558)
	2013	345(217–468)	168(95–240)	68(43–94)	7,880(5,962–9,721)	183(54–314)	414(252–573)	2,33220(1,1176–4,52456)
	2014	111(70–151)	55(31–79)	22(14–31)	2,755(2,080–3,405)	78(23–134)	515(314–713)	7,6980(3,673–1,49990)

	All-Cause Mortality	Cardiovascular Mortality	Respiratory Mortality	All-Caused Hospital Admission	Cardiovascular Impatient	Respiratory Impatient	ALL CAUSED Outpatient Visits
2015	320(201–434)	158(90–226)	63(40–87)	8,102(6,128–9,997)	304(89–521)	794(484–1,099)	2,20134(1,0543–4,27318)
2016	118(74–161)	61(34–87)	24(15–33)	3,285(2,480–4,061)	140(41–242)	705(429–978)	8,4981(4,056–1,65550)
Guigang	1,563(981–2,121)	750(426–1,072)	321(201–443)	3,6018(2,7220–4,4480)	1,185(347–2,035)	4,421(2,695–6,123)	1,059046(5,0639–2,059176)
2011	266(167–361)	122(69–174)	53(33–73)	4,655(3,518–5,749)	155(46–267)	614(374–850)	1,65523(7,914–3,21848)
2012	312(196–424)	138(78–197)	73(45–100)	6,381(4,824–7,878)	158(46–271)	591(360–818)	2,10106(1,0052–4,08279)
2013	205(129–278)	100(57–143)	41(25–56)	4,692(3,544–5,797)	109(32–187)	657(401–909)	1,38454(6,613–2,69491)
2014	163(102–221)	80(46–115)	32(20–45)	4,020(3,035–4,968)	114(33–195)	788(481–1,091)	1,12371(5,364–2,18866)
2015	349(219–474)	173(98–247)	69(43–95)	8,848(6,690–1,0922)	331(97–569)	893(544–1,237)	2,40264(1,1500–4,66706)
2016	268(168–363)	137(78–196)	54(34–74)	7,422(5,609–9,166)	318(93–546)	878(534–1,218)	1,92329(9,196–3,73986)
Qinzhou	1,289(809–1,749)	618(351–884)	265(166–365)	2,9614(2,2383–3,6566)	935(274–1,606)	2,709(1,649–3,756)	8,73599(4,1784–1,698099)
2011	206(130–280)	94(54–135)	41(26–56)	3,609(2,728–4,457)	121(35–207)	416(253–576)	1,28362(6,138–2,49553)
2012	252(158–342)	111(63–159)	59(37–81)	5,156(3,899–6,365)	127(37–219)	335(204–464)	1,69843(8,129–3,29915)
2013	183(115–249)	89(51–128)	36(23–50)	4,195(3,170–5,182)	97(29–167)	358(218–496)	1,23853(5,919–2,40928)
2014	273(171–370)	135(76–192)	54(34–75)	6,720(5,082–8,294)	190(56–327)	386(235–535)	1,88373(9,019–3,65801)
2015	190(119–258)	94(53–135)	38(23–52)	4,822(3,644–5,956)	180(53–310)	575(350–798)	1,30784(6,251–2,54396)
2016	184(116–250)	94(54–135)	37(23–51)	5,111(3,861–6,312)	219(64–376)	640(389–887)	1,32383(6,327–2,57505)
Laibing	1,222(768–1,658)	584(332–834)	251(157–345)	2,7592(2,0872–3,4044)	871(256–1,493)	2,067(1,259–2,864)	8,24396(3,9499–1,599714)
2011	252(158–342)	115(65–164)	50(31–68)	4,398(3,329–5,422)	147(43–252)	350(214–484)	1,56879(7,528–3,03966)
2012	234(147–317)	103(59–147)	54(34–75)	4,769(3,609–5,882)	118(35–202)	213(130–296)	1,57361(7,546–3,05108)
2013	204(128–277)	99(56–142)	40(25–56)	4,666(3,530–5,758)	108(32–186)	205(125–285)	1,38069(6,614–2,67953)
2014	197(124–267)	97(55–139)	39(25–54)	4,850(3,668–5,985)	137(40–236)	401(245–554)	1,35995(6,513–2,64001)
2015	164(103–223)	81(46–116)	32(20–45)	4,166(3,150–5,144)	156(46–268)	419(255–580)	1,13093(5,411–2,19763)
2016	171(107–232)	88(50–125)	34(22–48)	4,742(3,586–5,854)	203(60–349)	479(291–664)	1,22999(5,887–2,38923)
Wuzhou	1,211(761–1,644)	580(329–828)	246(154–339)	2,7229(2,0586–3,3612)	841(247–1,444)	2,347(1,429–3,256)	8,14078(3,8962–1,581416)
2011	282(177–383)	129(73–184)	56(35–77)	4,929(3,729–6,081)	165(48–283)	203(123–282)	1,75617(8,415–3,40752)
2012	170(107–231)	75(43–107)	40(25–55)	3,473(2,624–4,290)	86(25–147)	231(141–321)	1,14255(5,461–2,22255)
2013	158(99–215)	77(44–110)	31(20–43)	3,618(2,734–4,470)	84(25–144)	258(157–357)	1,06803(5,103–2,07819)
2014	335(211–455)	166(94–236)	67(42–92)	8,258(6,250–1,0184)	234(69–401)	500(305–692)	2,31851(1,1119–4,49463)
2015	183(115–248)	90(51–129)	36(23–50)	4,626(3,495–5,713)	173(51–297)	517(314–717)	1,25465(5,997–2,44027)
2016	84(52–114)	43(24–61)	17(11–23)	2,324(1,754–2,873)	99(29–171)	639(389–887)	6,0087(2,866–1,17100)
Hezhou	743(467–1,009)	358(203–513)	152(95–209)	1,7281(1,3059–2,1342)	547(160–939)	1,747(1,065–2,420)	5,04971(2,4142–9,81972)
2011	104(66–142)	48(27–68)	21(13–29)	1,828(1,381–2,259)	61(18–105)	126(76–175)	6,4970(3,104–1,26419)
2012	115(72–156)	51(29–73)	27(17–37)	2,350(1,776–2,903)	58(17–100)	142(86–198)	7,7309(3,695–1,50386)
2013	150(94–203)	73(41–104)	30(19–41)	3,421(2,586–4,223)	79(23–136)	166(101–231)	1,01093(4,837–1,96443)
2014	143(90–194)	71(40–101)	29(18–39)	3,529(2,667–4,357)	100(29–171)	523(320–721)	9,8830(4,727–1,92094)
2015	110(69–149)	54(31–78)	22(14–30)	2,784(2,103–3,439)	104(31–179)	386(235–535)	7,5468(3,606–1,46844)
2016	121(76–165)	62(35–89)	25(15–34)	3,370(2,546–4,162)	144(42–248)	404(246–561)	8,7303(4,174–1,69786)
Chongzuo	583(366–791)	277(157–397)	119(74–164)	1,2810(9,677–1,5825)	399(117–686)	1,663(1,012–2,307)	3,89155(1,8596–7,57151)
2011	175(110–238)	80(45–114)	35(22–48)	3,062(2,316–3,779)	102(30–176)	232(142–322)	1,09019(5,220–2,11666)
2012	92(58–125)	41(23–58)	21(13–30)	1,881(1,421–2,324)	46(14–80)	198(120–274)	6,1830(2,953–1,20370)
2013	95(60–129)	46(26–66)	19(12–26)	2,172(1,640–2,683)	50(15–87)	219(133–303)	6,4074(3,060–1,24729)
2014	101(64–137)	50(28–72)	20(13–28)	2,499(1,888–3,088)	71(21–121)	303(185–420)	6,9908(3,339–1,36064)
2015	54(34–73)	27(15–38)	11(7–15)	1,367(1,032–1,690)	51(15–88)	347(211–481)	3,6998(1,765–7,2117)
2016	66(41–90)	34(19–48)	13(8–18)	1,830(1,381–2,262)	78(23–135)	364(222–506)	4,7326(2,258–9,2206)

	All-Cause Mortality	Cardiovascular Mortality	Respiratory Mortality	All-Caused Hospital Admission	Cardiovascular Impatient	Respiratory Impatient	ALL CAUSED Outpatient Visits
Beihai	546(343–742)	261(148–373)	111(69–153)	1,2235(9,247–1,5108)	388(114–666)	1,136(691–1,577)	3,65953(1,7501–7,11451)
2011	132(83–179)	60(34–86)	26(16–36)	2,305(1,743–2,845)	77(23–132)	192(117–266)	8,2062(3,929–1,59351)
2012	75(47–102)	33(19–48)	18(11–24)	1,542(1,165–1,906)	38(11–65)	145(88–201)	5,0706(2,422–9,8699)
2013	96(60–131)	47(27–67)	19(12–26)	2,205(1,666–2,724)	51(15–88)	141(86–196)	6,5122(3,113–1,26659)
2014	65(41–88)	32(18–46)	13(8–18)	1,603(1,211–1,982)	45(13–78)	211(128–292)	4,4831(2,140–8,7305)
2015	146(92–199)	72(41–104)	29(18–40)	3,705(2,802–4,572)	139(41–238)	217(132–301)	1,00645(4,820–1,95395)
2016	31(20–43)	16(9–23)	6(4–9)	874(660–1,081)	37(11–64)	230(140–320)	2,2587(1,077–4,4043)
Fangchenggang	164(103–223)	77(43–110)	35(22–48)	3,525(2,662–4,356)	105(31–180)	667(405–925)	1,09506(5,229–2,13209)
2011	41(26–56)	19(11–27)	8(5–11)	726(548–897)	24(7–42)	120(73–166)	2,5789(1,232–5,0198)
2012	59(37–80)	26(15–37)	14(9–19)	1,198(906–1,480)	30(9–51)	85(52–118)	3,9435(1,886–7,6672)
2013	20(13–27)	10(6–14)	4(3–6)	463(349–572)	11(3–18)	92(56–127)	1,3641(650–2,6594)
2014	22(14–30)	11(6–15)	4(3–6)	537(405–664)	15(4–26)	107(65–148)	1,4999(715–2,9240)
2015	3(2–5)	2(1–2)	1(0–1)	87(66–108)	3(1–6)	129(79–180)	2,355(112–4,598)
2016	18(12–25)	9(5–14)	4(2–5)	514(388–636)	22(6–38)	134(81–186)	1,3287(634–2,5907)

Table S9. Adverse Health Effect caused by NO_2.

	All-Cause Mortality	Cardiovascular Mortality	Respiratory Mortality	All-Caused Hospital Admission	Cardiovascular Impatient	Respiratory Impatient	ALL CAUSED Outpatient Visits
Total	4,6365(3,1158–6,1423)	2,2172(1,2408–3,1675)	8,765(5,058–1,2366)	7,36973(5,98040–8,75052)	1,9037(2,395–3,5239)	3,8044(2,2837–5,3074)	2,2784,511(2,320249–2,7110,320)
Nanning	1,0367(6,979–1,3710)	4,977(2,793–7,092)	1,948(1,128–2,739)	1,66909(1,35600–1,97954)	4,354(549–8,041)	6,693(4,022–9,329)	5,122834(5,23233–6,091588)
2011	1,567(1,054–2,073)	713(400–1,017)	286(165–402)	1,9048(1,5470–2,2598)	523(66–966)	892(536–1,244)	7,09345(7,2391–8,43635)
2012	1,596(1,074–2,112)	702(394–1,001)	342(198–482)	2,2684(1,8423–2,6912)	461(58–851)	754(452–1,051)	7,81837(7,9788–9,29850)
2013	1,978(1,333–2,614)	958(538–1,364)	361(209–506)	3,1448(2,5564–3,7276)	602(76–1,110)	1,037(624–1,442)	9,74824(9,9718–1,158792)
2014	1,948(1,312–2,574)	958(538–1,364)	357(207–502)	3,3396(2,7144–3,9591)	779(98–1,437)	1,237(744–1,722)	9,81014(1,00317–1,166236)
2015	1,715(1,154–2,269)	846(475–1,206)	312(180–438)	3,0205(2,4534–3,5832)	930(117–1,718)	1,385(832–1,931)	8,58401(8,7623–1,020858)
2016	1,563(1,052–2,069)	799(448–1,139)	291(168–409)	3,0127(2,4465–3,5745)	1,061(134–1,960)	1,389(833–1,939)	8,17413(8,3397–9,72217)
Guilin	5,870(3,947–7,773)	2,803(1,570–4,002)	1,110(641–1,564)	9,2870(7,5382–1,10242)	2,402(302–4,444)	4,725(2,839–6,586)	2,882729(2,93776–3,429501)
2011	1,121(754–1,483)	510(286–728)	204(118–288)	1,3627(1,1067–1,6166)	374(47–691)	668(401–931)	5,07479(5,1793–6,03544)
2012	1,046(703–1,385)	460(258–657)	224(130–316)	1,4868(1,2071–1,7644)	302(38–558)	558(335–779)	5,12047(5,2212–6,09094)
2013	1,094(736–1,448)	530(297–757)	200(116–282)	1,7406(1,4134–2,0654)	332(42–614)	672(404–935)	5,38183(5,4895–6,40137)
2014	939(631–1,243)	462(259–660)	173(100–244)	1,6105(1,3070–1,9121)	374(47–692)	909(547–1,265)	4,71410(4,8012–5,60894)
2015	808(542–1,071)	399(223–570)	147(85–208)	1,4231(1,1543–1,6904)	436(55–808)	888(533–1,239)	4,03177(4,1011–4,79838)
2016	863(580–1,143)	441(247–630)	161(93–227)	1,6634(1,3497–1,9753)	584(73–1,082)	1,030(618–1,437)	4,50434(4,5853–5,35995)
Yulin	5,259(3,533–6,970)	2,539(1,420–3,629)	985(568–1,390)	8,5413(6,9300–1,01431)	2,248(283–4,162)	3,319(1,989–4,638)	2,595324(2,64158–3,088402)
2011	882(592–1,169)	402(225–575)	161(93–228)	1,0731(8,704–1,2747)	294(37–544)	442(265–618)	3,98471(4,0532–4,74238)
2012	563(378–748)	248(138–356)	121(70–172)	8,014(6,494–9,529)	162(20–300)	283(170–396)	2,74784(2,7877–3,27216)
2013	691(464–917)	335(187–480)	127(73–180)	1,0999(8,916–1,3074)	209(26–387)	284(170–397)	3,38755(3,4400–4,03311)
2014	1,519(1,023–2,007)	747(420–1,064)	279(162–392)	2,6039(2,1159–3,0875)	607(77–1,120)	603(361–842)	7,64476(7,8127–9,08929)
2015	789(529–1,046)	389(217–557)	144(83–204)	1,3896(1,1267–1,6513)	426(53–789)	814(488–1,137)	3,93324(3,9966–4,68217)
2016	816(548–1,082)	417(233–597)	152(88–215)	1,5733(1,2759–1,8693)	552(69–1,023)	893(535–1,248)	4,25514(4,3256–5,06490)
Liuzhou	4,627(3,111–6,127)	2,203(1,234–3,146)	877(507–1,236)	7,2748(5,9050–8,6353)	1,850(233–3,423)	3,914(2,352–5,454)	2,269710(2,31329–2,700147)
2011	883(594–1,168)	402(225–573)	161(93–227)	1,0732(8,716–1,2732)	295(37–544)	516(310–719)	3,99641(4,0784–4,75299)

		All-Cause Mortality	Cardiovascular Mortality	Respiratory Mortality	All-Caused Hospital Admission	Cardiovascular Impatient	Respiratory Impatient	ALL CAUSED Outpatient Visits
	2012	904(609–1,197)	398(223–567)	194(112–273)	1,2853(1,0439–1,5248)	261(33–482)	450(270–627)	4,43027(4,5215–5,26892)
	2013	853(574–1,129)	413(232–590)	156(90–220)	1,3567(1,1016–1,6100)	259(33–478)	566(341–788)	4,19443(4,2778–4,98917)
	2014	822(553–1,088)	405(227–578)	151(87–213)	1,4103(1,1449–1,6738)	328(41–606)	780(469–1,084)	4,13127(4,2115–4,91452)
	2015	587(394–778)	290(162–415)	107(62–151)	1,0342(8,387–1,2288)	317(40–587)	746(448–1,040)	2,92847(2,9769–3,48576)
	2016	578(388–767)	296(165–423)	108(62–152)	1,1151(9,043–1,3247)	391(49–725)	857(514–1,195)	3,01625(3,0668–3,59010)
Guigang		3,912(2,627–5,186)	1,871(1,046–2,676)	740(427–1,046)	6,2176(5,0431–7,3858)	1,597(201–2,960)	3,619(2,173–5,048)	1,920802(1,95368–2,286064)
	2011	715(480–947)	326(182–465)	131(75–185)	8,693(7,053–1,0324)	238(30–441)	502(302–701)	3,22962(3,2870–3,84326)
	2012	674(452–893)	297(166–424)	145(84–205)	9,580(7,771–1,1380)	194(24–359)	484(291–675)	3,29234(3,3489–3,91836)
	2013	631(424–837)	306(171–438)	116(67–164)	1,0042(8,144–1,1931)	191(24–354)	538(324–750)	3,09602(3,1476–3,68511)
	2014	855(575–1,132)	421(236–601)	157(91–222)	1,4669(1,1906–1,7413)	341(43–630)	646(388–900)	4,29539(4,3767–5,11027)
	2015	488(328–648)	241(135–346)	89(51–126)	8,607(6,976–1,0232)	263(33–489)	731(439–1,020)	2,43393(2,4705–2,89804)
	2016	549(368–728)	281(157–402)	102(59–145)	1,0584(8,581–1,2578)	371(47–688)	718(430–1,003)	2,86071(2,9061–3,40560)
Hechi		3,122(2,096–4,140)	1,497(837–2,142)	594(342–840)	5,0488(4,0949–5,9977)	1,330(167–2,465)	2,700(1,620–3,769)	1,542376(1,56850–1,835746)
	2011	420(282–557)	191(107–274)	77(44–109)	5,107(4,140–6,070)	139(18–259)	372(223–519)	1,89379(1,9233–2,25464)
	2012	633(425–838)	278(156–398)	136(78–192)	8,994(7,299–1,0679)	182(23–337)	343(206–478)	3,09388(3,1504–3,68133)
	2013	489(328–648)	237(132–339)	90(52–127)	7,781(6,309–9,245)	148(19–274)	339(203–472)	2,39813(2,4373–2,85462)
	2014	518(348–687)	255(143–365)	96(55–135)	8,897(7,215–1,0570)	206(26–382)	421(253–588)	2,60019(2,6435–3,09493)
	2015	470(315–623)	232(130–332)	86(49–121)	8,282(6,715–9,842)	254(32–470)	650(390–906)	2,34380(2,3812–2,79017)
	2016	593(398–785)	303(170–433)	110(64–156)	1,1427(9,271–1,3570)	401(50–743)	576(345–805)	3,09396(3,1491–3,68176)
Qinzhou		2,590(1,738–3,435)	1,234(689–1,766)	493(284–698)	4,0919(3,3179–4,8624)	1,037(130–1,923)	2,216(1,329–3,094)	1,270072(1,29076–1,511855)
	2011	439(295–583)	200(112–286)	80(46–114)	5,344(4,333–6,350)	146(18–271)	340(204–475)	1,98293(20154–2,36038)
	2012	544(366–721)	240(134–342)	117(67–165)	7,739(6,279–9,190)	157(20–290)	274(164–382)	2,66082(2,7081–3,16638)
	2013	491(330–651)	238(133–341)	90(52–127)	7,814(6,338–9,283)	149(19–275)	293(176–409)	2,40959(2,4504–2,86792)
	2014	437(293–580)	215(120–308)	81(46–114)	7,504(6,084–8,917)	174(22–322)	315(189–440)	2,19187(2,2272–2,60923)
	2015	335(225–445)	166(92–237)	61(35–87)	5,908(4,787–7,024)	181(23–335)	471(282–657)	1,66993(1,6944–1,98852)
	2016	343(230–455)	175(98–251)	64(37–91)	6,612(5,358–7,860)	231(29–429)	523(313–731)	1,78558(1,8122–2,12612)
Baise		2,433(1,633–3,228)	1,163(649–1,664)	462(266–654)	3,8686(3,1367–4,5973)	948(119–1,758)	2,982(1,791–4,159)	1,194947(1,21408–1,422511)
	2011	349(234–464)	159(89–228)	64(37–91)	4,249(3,443–5,052)	116(15–215)	344(207–481)	1,57417(1,5970–1,87454)
	2012	460(308–610)	202(113–290)	99(57–140)	6,538(5,301–7,770)	132(17–245)	259(155–362)	2,24426(2,2798–2,67174)
	2013	417(279–553)	202(113–290)	77(44–108)	6,632(5,375–7,883)	126(16–233)	299(179–418)	2,04203(2,0732–2,43130)
	2014	730(491–966)	359(201–513)	134(78–189)	1,2517(1,0160–1,4858)	291(37–538)	719(433–999)	3,66575(3,7357–4,36104)
	2015	286(191–380)	141(79–202)	52(30–74)	5,037(4,080–5,992)	154(19–286)	655(393–914)	1,42257(1,4418–1,69437)
	2016	192(129–256)	98(55–141)	36(21–51)	3,713(3,007–4,418)	130(16–241)	706(424–986)	1,00069(1,0132–1,19214)
Wuzhou		2,332(1,566–3,092)	1,110(620–1,588)	440(253–622)	3,6170(2,9332–4,2974)	954(120–1,768)	1,920(1,151–2,682)	1,134837(1,15385–1,350743)
	2011	622(418–824)	283(159–404)	114(66–160)	7,566(6,143–8,979)	208(26–384)	166(99–232)	2,81564(2,8710–3,34928)
	2012	367(246–487)	162(90–231)	79(45–112)	5,220(4,232–6,204)	105(13–196)	189(113–264)	1,79150(1,8195–2,13285)
	2013	491(330–650)	238(133–340)	90(52–127)	7,807(6,333–9,273)	148(19–275)	211(126–294)	2,40855(2,4505–2,86639)
	2014	123(82–164)	61(34–87)	23(13–32)	2,115(1,712–2,518)	49(6–91)	409(246–571)	6,1563(6,230–7,3350)
	2015	358(240–475)	177(99–253)	65(38–92)	6,303(5,109–7,492)	193(24–358)	423(253–590)	1,78264(1,8099–2,12245)
	2016	371(249–492)	190(106–272)	69(40–98)	7,158(5,803–8,507)	251(31–465)	523(313–730)	1,93441(1,9648–2,30295)
Laibing		2,099(1,410–2,782)	996(557–1,424)	401(231–566)	3,2931(2,6715–3,9113)	845(106–1,565)	1,691(1,015–2,360)	1,028543(1,04670–1,223996)
	2011	388(261–514)	177(99–253)	71(41–100)	4,723(3,833–5,608)	129(16–239)	287(172–400)	1,75552(1,7876–2,08885)
	2012	503(338–665)	221(124–315)	108(62–152)	7,145(5,803–8,476)	145(18–268)	175(105–244)	2,46256(2,5132–2,92874)
	2013	283(190–375)	137(77–196)	52(30–73)	4,498(3,647–5,346)	85(11–158)	168(101–234)	1,38589(1,4079–1,64987)

	All-Cause Mortality	Cardiovascular Mortality	Respiratory Mortality	All-Caused Hospital Admission	Cardiovascular Impatient	Respiratory Impatient	ALL CAUSED Outpatient Visits
2014	344(231–456)	169(95–242)	63(37–90)	5,900(4,786–7,009)	137(17–253)	328(197–457)	1,72494(1,7544–2,05298)
2015	329(221–436)	162(91–232)	60(35–85)	5,793(4,698–6,883)	178(22–329)	342(205–478)	1,64048(1,6678–1,95262)
2016	253(169–335)	129(72–185)	47(27–67)	4,871(3,949–5,790)	171(21–316)	392(235–547)	1,31605(1,3362–1,56691)
Chongzuo	1,419(952–1,882)	673(376–964)	267(154–378)	2,1647(1,7550–2,5726)	568(71–1,053)	1,360(816–1,900)	6,86354(6,9745–8,17037)
2011	469(315–620)	213(120–304)	86(49–120)	5,700(4,629–6,762)	156(20–289)	190(114–266)	2,12254(2,1661–2,52436)
2012	199(133–264)	88(49–126)	43(25–61)	2,829(2,292–3,364)	57(7–106)	162(97–226)	9,6994(9,839–1,15504)
2013	191(128–254)	93(52–133)	35(20–50)	3,048(2,469–3,624)	58(7–107)	179(107–250)	9,3755(9,509–1,11652)
2014	204(137–271)	101(56–144)	38(22–53)	3,505(2,840–4,168)	81(10–150)	248(149–346)	1,02238(1,0372–1,21748)
2015	177(119–236)	88(49–126)	32(19–46)	3,126(2,533–3,718)	96(12–177)	284(170–396)	8,8314(8,954–1,05180)
2016	178(119–237)	91(51–131)	33(19–47)	3,439(2,786–4,090)	120(15–223)	298(178–416)	9,2799(9,410–1,10518)
Hezhou	1,214(814–1,612)	577(322–827)	232(133–328)	1,8952(1,5359–2,2533)	476(60–883)	1,430(858–1,995)	5,92677(6,0149–7,05714)
2011	255(171–338)	116(65–166)	47(27–66)	3,101(2,514–3,685)	85(11–157)	103(61–144)	1,14997(1,1682–1,36902)
2012	248(167–330)	109(61–157)	53(31–76)	3,532(2,863–4,198)	71(9–132)	116(70–163)	1,21219(1,2311–1,44317)
2013	197(132–262)	96(53–137)	36(21–51)	3,140(2,545–3,734)	60(7–111)	136(82–190)	9,6631(9,802–1,15071)
2014	254(170–337)	125(70–179)	47(27–66)	4,359(3,534–5,181)	101(13–187)	429(258–596)	1,27288(1,2927–1,51541)
2015	117(78–155)	58(32–83)	21(12–30)	2,055(1,664–2,445)	63(8–117)	316(189–441)	5,7992(5,872–6,9086)
2016	143(96–190)	73(41–105)	27(15–38)	2,764(2,239–3,288)	97(12–179)	330(198–462)	7,4550(7,554–8,8798)
Beihai	622(417–826)	291(162–418)	120(69–170)	9,305(7,538–1,1068)	236(30–438)	929(557–1,298)	2,99642(3,0376–3,56877)
2011	193(129–256)	88(49–126)	35(20–50)	2,346(1,901–2,788)	64(8–119)	157(94–219)	8,6974(8,833–1,03546)
2012	163(109–216)	72(40–103)	35(20–50)	2,319(1,880–2,758)	47(6–87)	119(71–166)	7,9537(8,070–9,4710)
2013	57(38–76)	28(15–40)	10(6–15)	904(732–1,076)	17(2–32)	115(69–161)	2,7751(2,807–3,3066)
2014	81(54–108)	40(22–57)	15(9–21)	1,391(1,126–1,655)	32(4–60)	172(103–241)	4,0488(4,099–4,8236)
2015	77(52–103)	38(21–55)	14(8–20)	1,360(1,101–1,618)	41(5–77)	177(106–248)	3,8356(3,882–4,5698)
2016	51(34–68)	26(15–38)	10(5–14)	986(798–1,173)	34(4–64)	188(113–263)	2,6537(2,684–3,1620)
Fangchenggang	498(334–662)	236(132–338)	96(55–136)	7,759(6,288–9,225)	191(24–355)	545(327–762)	2,43663(2,4727–2,90140)
2011	90(60–120)	41(23–59)	17(10–23)	1,098(889–1,305)	30(4–56)	98(59–137)	4,0663(4,126–4,8420)
2012	127(85–168)	56(31–80)	27(16–39)	1,800(1,460–2,139)	36(5–67)	69(42–97)	6,1814(6,283–7,3580)
2013	115(77–152)	56(31–80)	21(12–30)	1,825(1,479–2,169)	35(4–64)	75(45–105)	5,6213(5,709–6,6922)
2014	66(44–88)	33(18–47)	12(7–17)	1,134(918–1,349)	26(3–49)	87(52–122)	3,3035(3,347–3,9349)
2015	28(19–37)	14(8–20)	5(3–7)	494(400–588)	15(2–28)	106(63–148)	1,3926(1,408–1,6594)
2016	73(49–97)	37(21–54)	14(8–19)	1,409(1,141–1,676)	49(6–91)	110(66–153)	3,8013(3,853–4,5274)
